# METHODOLOGY FOR ANALYZING MEDICAID NURSING HOME PAYMENT RATES AND COSTS

**DOI:** 10.1093/geroni/igae098.2003

**Published:** 2024-12-31

**Authors:** Elizabeth Simpson, John Bowblis, Edward Miller, Sara Karon, Iara Oliveira, Judith Dey, Marie Squillace, Marc Cohen

**Affiliations:** University of Massachusetts Boston, Boston, Massachusetts, United States; Miami University, Oxford, Ohio, United States; University of Massachusetts Boston, Boston, Massachusetts, United States; RTI International, Research Triangle Park, North Carolina, United States; Office of Behavioral Health, Disability, and Aging Policy (BHDAP), Washington, District of Columbia, United States; US Department of Health and Human Services, Washington, District of Columbia, United States; Office of the Assistant Secretary for Planning and Evaluation, Washington, District of Columbia, United States; University of Massachusetts Boston, Boston, Massachusetts, United States

## Abstract

This study required the construction of a unique facility-by-facility national database that links Medicaid nursing home (NH) payment, cost, staffing, and quality information from a variety of sources to enable detailed analysis of key financial and other metrics. Medicaid cost data were obtained from Medicare Cost Reports and Medicaid payment rates were constructed from statutory rates collected from the states. Medicaid costs were adjusted to capture only services provided to Medicaid residents. The final sample consisted of 9,543 NHs, representing 67% of all freestanding NHs in 2019 in the 44 states analyzed. The analysis focused on distributions, means, medians, and payment-to-cost ratios, where a ratio of 1.0 indicates that Medicaid payments matched reported NH costs. The impacts of NH characteristics and state payment methods on Medicaid costs, payments, and payment-to-cost ratios were explored. Regression analyses further investigated the impact of NH and state policy characteristics, accounting for state fixed effects.

